# Artificial Intelligence-Based Smart Quality Inspection for Manufacturing

**DOI:** 10.3390/mi14030570

**Published:** 2023-02-27

**Authors:** Sarvesh Sundaram, Abe Zeid

**Affiliations:** College of Engineering, Northeastern University, Boston, MA 02135, USA

**Keywords:** artificial intelligence, deep learning, quality control, visual inspection, industry 4.0, smart manufacturing, image recognition, defect detection

## Abstract

In today’s era, monitoring the health of the manufacturing environment has become essential in order to prevent unforeseen repairs, shutdowns, and to be able to detect defective products that could incur big losses. Data-driven techniques and advancements in sensor technology with Internet of the Things (IoT) have made real-time tracking of systems a reality. The health of a product can also be continuously assessed throughout the manufacturing lifecycle by using Quality Control (QC) measures. Quality inspection is one of the critical processes in which the product is evaluated and deemed acceptable or rejected. The visual inspection or final inspection process involves a human operator sensorily examining the product to ascertain its status. However, there are several factors that impact the visual inspection process resulting in an overall inspection accuracy of around 80% in the industry. With the goal of 100% inspection in advanced manufacturing systems, manual visual inspection is both time-consuming and costly. Computer Vision (CV) based algorithms have helped in automating parts of the visual inspection process, but there are still unaddressed challenges. This paper presents an Artificial Intelligence (AI) based approach to the visual inspection process by using Deep Learning (DL). The approach includes a custom Convolutional Neural Network (CNN) for inspection and a computer application that can be deployed on the shop floor to make the inspection process user-friendly. The inspection accuracy for the proposed model is 99.86% on image data of casting products.

## 1. Introduction

The manufacturing and production industry has undergone a fundamental transformation over the past century. Strategies such as Total Quality Management (TQM), Six Sigma, Lean, and Zero-Defect Manufacturing have pushed for higher yields while lowering costs. More recently, approaches such as Industry 4.0, Cyber-Physical Systems and Smart Manufacturing are allowing an interconnected shop floor environment with developments in robotics and automation. While larger enterprises have the economic prowess to implement transformative changes to the industry, Small and Medium-sized Enterprises (SMEs) are often unable to do so. When it comes to overall Quality Management (QM), SMEs face challenges due to the following reasons: there is a lack of information about the importance of QM, they face resource constraints, and there is an inadequacy in standards for SMEs [1,2]. Artificial Intelligence (AI) is tackling this with the development of novel algorithms, and with the help of low-cost sensors and computing services. The capabilities of the manufacturing paradigm are being enhanced by Machine Learning (ML), Pattern Recognition (PR), and Deep Learning (DL). Applications of AI are increasingly enabling manufacturers across the board to identify faulty components in their systems, detect defective products, and in some instances, diagnose them. On the system side, the area of Prognostics and Health Management (PHM) is transforming approaches to maintenance. On the product side, AI helps in Quality Control (QC) measures. While these systems are modernizing many of the manufacturing processes, there still seems to be a reliance on human operators for some critical decision-making steps on the shop floor. Quality inspection is one such process.

Quality inspection is a planned and organized process in which the state of the product is assessed by examination, measurement, testing, gauging, or comparison to determine if it conforms to desired specifications [3]. In most cases, quality inspection involves a human operator that inspects the product to ascertain its conformity. However, the accuracy and reliability of the inspection are often unsatisfactory. According to Harris [4], as the complexity of the product increases, the accuracy of inspection conducted by operators decreases. Similarly, in a study conducted by the Sandia National Labs [5], the accuracy of correctly rejecting precision manufactured parts by human operators was found to be 85% while the industry average was 80%. Another recent study concluded that operator errors accounted for 23% of the inaccuracies in quality control in the oil and gas industry [6]. Over the years, computer vision-based systems have been incorporated into the inspection process of various products such as disk heads, steel strips [7], syringes [8] and semiconductors [9]. Vision-based systems generally consist of an algorithm that is taught to identify discrepancies between features of the product undergoing inspection and the desired features. Although these systems help in automating the inspection process to a certain extent, there are still some challenges to their implementation on the shop floor [10]. There are also numerous works in which ML and DL-based models are applied to the quality inspection process. However, a lot of researchers focus on improving the performance of models and do not consider a holistic approach to inspection. While that is one of the main goals of the inspection process, there are several factors that affect the inspection process that go unaddressed. There is also a need for a methodology or approach that establishes how a data-driven method can be deployed on the shop floor in a user-friendly and hassle-free manner.

To that end, this paper proposes a twofold approach to the quality inspection process. First, a DL algorithm based on a custom Convolutional Neural Network (CNN) architecture is constructed for defect detection, and then a shop floor tool is designed to deploy the inspection model. The rest of the paper is structured as follows. Section 2 discusses the role of health monitoring in manufacturing from the perspective of the system and the product. Section 3 reviews the state of quality inspection and visual inspection, and outlines some of the key factors that affect the inspection process. Section 4 provides an overview of the casting operation in manufacturing and the challenges it presents in the inspection process. Section 5 discusses the role of DL in the inspection and how DL has automated some of the steps in quality inspection. Section 6 highlights the state-of-the-art in visual inspection, identifies the research gap and objectives of our work, and Section 7 describes the research process and proposes a methodology for AI-based Smart Quality Inspection. Section 8 describes the casting product data used in this research. Section 9 explains the modelling of the algorithm and the design of the shopfloor application. Section 10 presents the results of Smart Quality Inspection on casting products. Section 11 provides concluding remarks and outlines the objectives of future work.

## 2. Health Monitoring in Manufacturing

**Figure 1:**
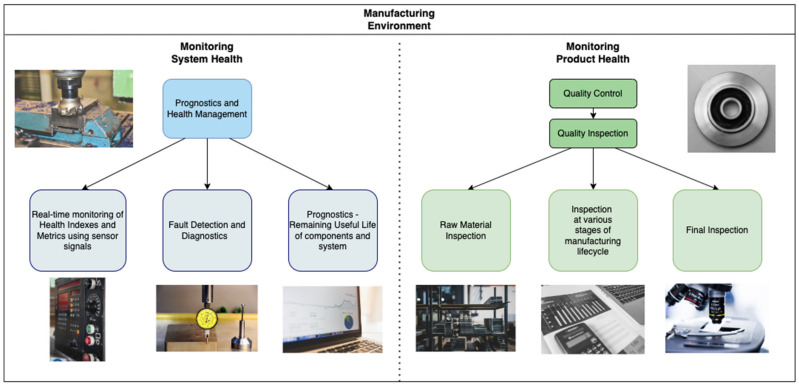
Health Monitoring of the manufacturing environment with PHM and QC.

It would be remiss to discuss quality inspection, a crucial component of the QC process, without providing a context about the health of the entire manufacturing environment. There are two major areas of health monitoring in manufacturing. The first concerns the system’s health, ensuring that the machinery and equipment are functioning satisfactorily. The second area pertains to monitoring the product’s health throughout its lifecycle. For the system, PHM deals with component-level and system-level health monitoring. On the product side, QC techniques are relied upon to guarantee the health and quality of the product. Figure 1 portrays the different phases of PHM and QC in the context of health monitoring in manufacturing. The rest of this section presents brief overviews of these topics.

### 2.1. Prognostics and Health Management (PHM)

Prognostics and Health Management (PHM) is a discipline that monitors the system’s health, detects failures, diagnoses failures, and predicts the Remaining Useful Life (RUL) of components [11]. Using the Internet of Things (IoT) -powered sensors and field devices, operating conditions of critical tools and components can be monitored in real-time. Nowadays, the availability of low-cost embedded devices and microcontrollers such as Raspberry Pi, STM32, Arduino, etc. enable SMEs to incorporate PHM on the shop floor. Once sufficient data has been collected, the health indexes and metrics developed can be used in models to predict failures of components and provide Remaining Useful Life (RUL) estimates. In the past, data-driven approaches to PHM have relied on ML models. However, successful implementations of ML models for prognostics or failure detection are often reliant upon expert knowledge to extract meaningful characteristics or features from the data [12]. DL techniques have the capabilities to automatically extract high-level features from inputs such as acoustic signals, vibration signals, image data, etc. Hence, there is an advantage to using DL for prognostics and diagnostics applications. A detailed methodology developed in [11] reviews the various approaches to PHM: data-driven, physics-based, and hybrid and use-case on health monitoring of a milling machine tool.

### 2.2. Quality Control (QC)

With the rise in modern manufacturing systems and the development of highly complex products, quality management has taken an essential role in organizational planning and strategies [13]. Quality Control (QC) is a process that involves setting quality standards, ensuring that the product meets those standards, and improving the overall product quality. Quality Inspection is a part of the QC process in which the product is inspected by operators during the various stages of manufacturing. Figure 1 depicts the QC process from the perspective of health monitoring, which involves product inspection. The overall QC process is continuously changing due to the dynamic nature of the manufacturing environment. Approaches such as Design of Experiments (DoE), Failure Mode and Effects Analysis (FMEA), Quality Function Deployment (QFD) and Acceptance Sampling prescribe their own methodologies for product inspection. While these approaches have been very successful in QC, there is an opportunity to assess 100% of the products on the modern shop floor. Continuous assessment of product quality has become a reality with developments in sensor networks and AI.

## 3. Overview of Quality Inspection

### 3.1. Quality Inspection Process

The traditional quality improvement process is cyclical—it involves generating inspection plans, implementing the plans, and checking the results [14]. Similarly, the inspection process is comprised of inspection plans that identify the different areas of manufacturing where inspection is required. It typically begins with the inspection of raw materials—also known as incoming or receiving inspection. Then there are inspections conducted periodically after various operations. The nature of these inspections is industry-specific in most cases. For instance, the inspection of structural steel products would differ greatly from the inspection of microcontrollers. At the end of the assembly line, a final inspection is conducted—where it is determined whether the product is acceptable or is to be rejected. This is analogous to outgoing inspection. In some cases, outgoing inspection refers to the inspection of the packaged product during shipping.

The inspection process is an important decision process in the manufacturing/production system [15]. According to the Signal Detection Theory (SDT), probabilistic decisions are made at every step by the decision maker (operator) to determine whether the product is to be accepted or rejected [16]. Inspection is not an independent process in the manufacturing value chain but impacts many other operations. The decision-making process for inspection involves multiple elements and should display the following characteristics as noted by [15]:

1. Precision: The decisions made should be well-informed, to ensure that there are no biases or errors.
2. Validity: Decisions made must be valid and must not differ if the product were to be available for use.
3. Reliability: There must be consistency in the decisions made—repeatability and reproducibility. The decision process should not require recalibration.
4. Robustness: The decision-making must demonstrate versatility in detecting different types of defects.
5. Rapidness: The process must be quick and must be able to act before any more defective products are produced.

Note that the above characteristics are desired from all inspection processes regardless of whether it is conducted by either human operators or by some form of automation.

### 3.2. Visual Inspection

An important type of quality inspection in manufacturing is visual inspection. Operators visually assess the state of the product at different stages and decide whether it can be moved on to the next process. Sinclair [17] suggested a four-step visual inspection operation comprising the following tasks:

1. Present: Present the product for inspection.
2. Search: Examine and analyze the product for possible flaws/defects.
3. Decision: Assess the flaws/defects and determine if it falls out of the desired specifications.
4. Action: Accept or reject the item based on the decision.

Similarly, Wang and Drury [18] characterized the visual inspection process as having a number of sub-tasks or activities: (1) orient the item, (2) search the item, (3) detect the defects/flaws, (4) recognize and classify the flaws/defects, (5) make a decision about the item, (6) dispatch the item, and (7) record any information about the item. In both approaches, the goal of the visual inspection process is to identify defects efficiently and accurately and make decisions accordingly.

### 3.3. Factors Affecting Visual Inspection

Any inspection process or system requires some form of human action. There cannot be a system that is entirely automated or manual [15]. Inspection involves a lot of mental effort, attention to detail, communication, and the usage of long-term and short-term memory [19]. In most cases, inspection is also required to be done quickly, i.e., defects must be identified swiftly before a decision is made. With human involvement, there arise several factors that could affect or impede the efficient implementation of visual inspection. According to research conducted by Peters et al. [20] and See et al. [21], some of the known factors that impact inspection can be categorized into task factors, environmental actors, operator or individual factors, organizational factors, and social factors.

Task factors refer to the manual and physical aspects of the inspection task. The task itself can affect the operator and influence their performance. Environmental factors can also significantly impact the outcome of visual inspection. Factors such as temperature, humidity, lighting, etc. can make the environment unsuitable which in turn influences the operator’s ability to conduct the inspection. Operator or individual factors refer to features such as an operator’s physical and mental attributes. Physical attributes could be an operator’s vision, visual acuity, gender, etc. Mental attributes could be their state of mind, aptitude, personality, biases, etc. Organizational factors concern the administration and management under which the inspection process takes place. It also includes the organizational importance given to quality inspection and visual inspection, training provided, etc. Social factors include relationships that the operator has with their peers and management, whether communication in their working environment is effective or not, and the other aspects of the social environment in which the inspection task occurs. A synopsis of all the factors that affect the visual inspection process based on [20,21] is provided in Table 1.

## 4. Casting Process

The manufacturing process of casting usually involves pouring liquefied metal into the cavity of a mold that is of the desired shape [22]. There are different types of casting processes in manufacturing. The type of process is dependent on the materials (mostly metals) used to manufacture the final product.

### 4.1. Types of Casting Processes

Some of the types of casting processes are listed with the types of materials that are used [23]:

(a) Sand Casting—most metal types
(b) Investment Casting—most metal types
(c) Resin Shell Molding—Primarily Iron and Copper
(d) Gravity Die Casting—Primarily Aluminum, Zinc, Magnesium, Copper, and some of their alloys
(e) Low-Pressure Die Casting—Primarily Aluminum and Magnesium
(f) High-Pressure Die Casting—Primarily Aluminum, Magnesium, and Zinc
(g) Squeeze Casting—Primarily Aluminum

### 4.2. Steps in the Casting Process

Most of the casting processes listed in Section 4.1 generally follow similar steps to go from the raw material to the finished product. A list of these steps is as follows [22]:

1. Patternmaking—Designing and preparing a pattern
2. Preparing the mold that is approximately the same shape/size as the desired pattern
3. Identifying the material to be used in casting (usually metals or allows)
4. Liquefying the material in a furnace
5. Pouring the liquefied metal into the cavity of the mold
6. Opening the mold to access the casting
7. Fettling—removing excess material, surface cleaning, and finishing
8. Heat treatment based on requirements
9. Final inspection

### 4.3. Inspection in the Casting Process

The quality inspection of casting products is the most critical step in determining whether the product is acceptable for use or must be rejected and scrapped/reworked. There are a few types of inspections for casting products: visual inspection, dimensional inspection, metallurgical inspection, chemical and physical inspection, and other methods involving Non-Destructive Testing (NDT) [24]. While multiple inspection methods are used concurrently during different stages of the casting process, we shall limit the discussion to a brief on visual inspection methods.

This visual inspection process for casting generally involves the examination of the product by an operator or a group of operators. Operators look for surface defects, cracks, tears, molding flaws, scabs, blowholes, runouts, adhesions, and various other types of defects [25]. Many of the defects can be attributed to flaws in mold design, the incorrect composition of materials used in mold construction, the equipment used in pouring liquefied metals into the molds, etc. Some visual inspection processes for casting products can be automated. Vision-based inspection systems rely on software for color matching and in some instances contour matching and dimension checking [26]. In recent years, ML and DL techniques have been used to perform visual inspections of casting products.

## 5. Deep Learning for Quality Inspection

Owing to the numerous factors that can affect an operator during the visual inspection process, data-driven approaches are being used increasingly to detect defective products. While traditional ML methods often require domain knowledge in the feature generation or feature engineering process, DL methods can automatically select and learn abstract features [27]. DL methods based on CNN, Autoencoders, and Recurrent Neural Networks (RNN) provide excellent results on a variety of inspection applications [28].

Chang et al. [29] apply a deep ensemble learning model to inspect defects on car body surfaces. Their method outperforms human inspectors in performing the same task. Researchers in [30] use a CNN to identify defects in textured surfaces. Results show high accuracy of defect detection on a multi-class dataset. For defect identification in semiconductor manufacturing, Imoto et al. [31] use a transfer learning approach based on CNN while Lee et al. [32] propose a CNN model that is receptive to time series data. In the inspection of sewer systems, Kumar et al. [33] propose deep CNNs. They use image data with high variation and claim that the CNN-based methods outperform other methods requiring manual feature extraction. For the inspection of laser welding, Yang et al. [34] use an optimized VGG model. A transfer learning approach is used where the VGG model is pre-trained on a large variety of images. Ullah et al. [35] propose an approach that uses a pre-trained AlexNet for feature extraction combined with Random Forest (RF) and Support Vector Machines (SVM) for defect detection. Their proposed method outperforms LeNet and VGG algorithms in an experiment conducted on hig- voltage electrical equipment. To inspect rivet joints in aircraft products, Amosov et al. [36] apply YOLOv5 and MobileNetv3 to images. In binary classification and in the multi-class scenario, they achieve very high accuracy of defect detection. To inspect the gas lighter manufacturing process, researchers in [37] develop a DL model based on YOLOv4. Results show good performance in detecting defects with changing illuminance and distance.

## 6. State-of-the-Art in Visual Inspection and Research Gap

There are several works that have implemented image-based quality inspection methods for defect detection in manufacturing products. He et al. [38] use CNN for defect detection on product surfaces at the pixel level. A convolutional variational autoencoder is proposed by Yun et al. [39] to study a multi-class surface defect identification problem on metals. In the case of welding products, Sassi et al. [40] apply a transfer learning approach and achieve a good performance on a small dataset. For inspection of casting products, Oborski et al. [41] use a CNN model in a holonic shopfloor based setting. In detecting defects of welded nuts, Lee et al. [42] use a model based on VGG-16. They conducted experiments with CNN models before achieving the desired performance with the VGG-16 model. Some more state-of-the-art defect detection methods in manufacturing are summarized in Table 2.

These research works achieve good performance on real-world data but do not provide a holistic approach to deploying DL-based models to the shop floor. While most of the works that apply AI to visual inspection focus on improving the model performance, very few of them take into consideration the various factors involved in the visual inspection process. An even fewer attempt to minimize these factors in their proposed methods. Factors outlined in Section 3.3 are crucial considerations while designing a visual inspection system. An inspection system should attempt to minimize the maximum number of the factors out of the task, environmental, operator, organizational, and social factors. Additionally, we have learned that even with automation, the inspection process will involve some form of human participation. Based on these arguments, there is a need for a visual inspection system that

1. performs well in detecting defects, i.e., shows high accuracy, precision, recall, etc.,
2. minimizes the factors affecting the visual inspection process, and
3. allows documentation of decisions made.

Considering the above requirements, we propose Smart Quality Inspection—an AI-based approach to the visual inspection process and demonstrate a use case on a benchmark image dataset from a casting process.

## 7. Smart Quality Inspection

**Figure 2:**
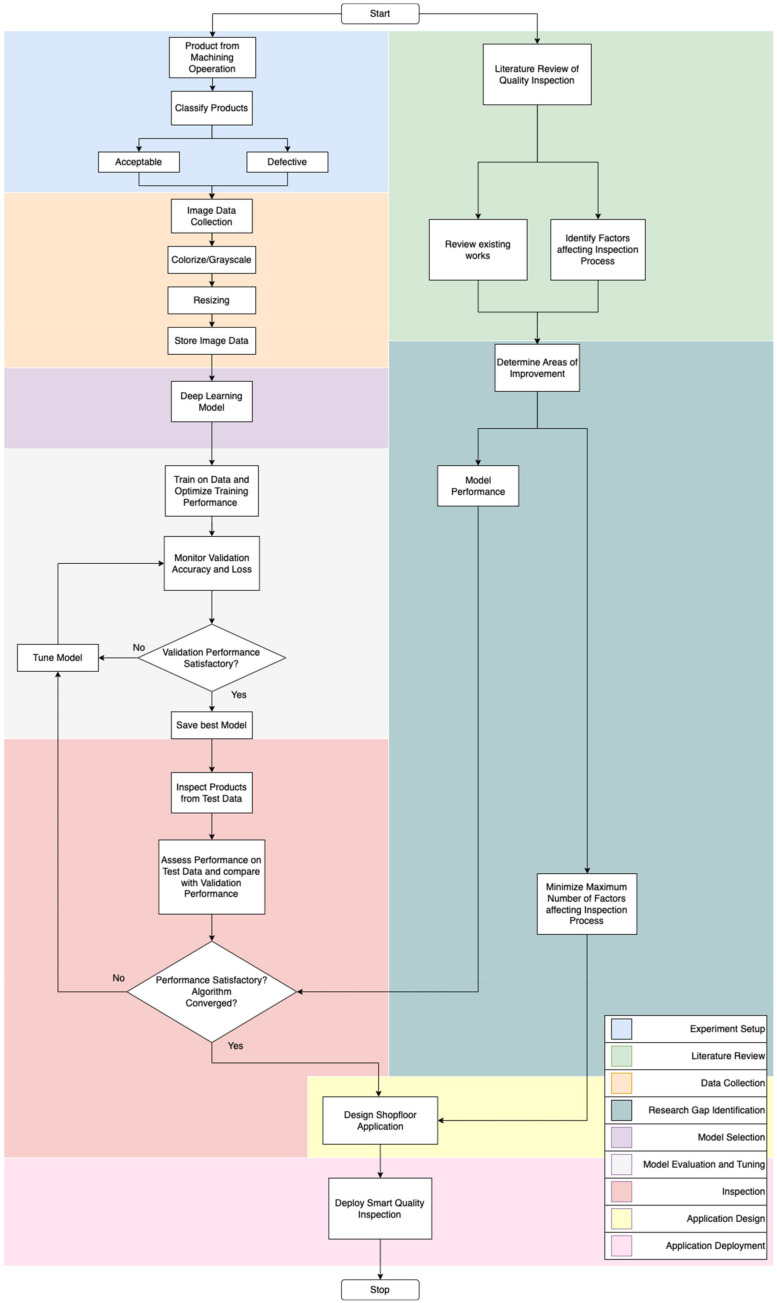
Flowchart depicting the process used to develop Smart Quality Inspection—algorithm construction and identification of research gap via literature review.

**Figure 3:**
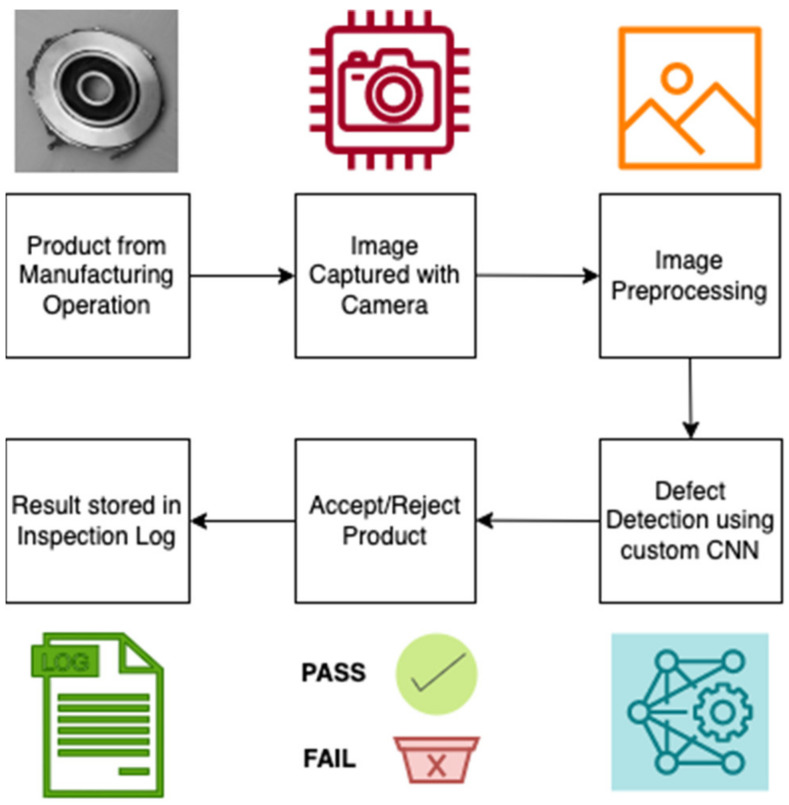
Artificial Intelligence based Smart Quality Inspection Methodology.

The Smart Quality Inspection (SQI) approach aims to improve model performance and address several factors that affect the visual inspection process. By automating the inspection process to an extent, the effects of many of the task factors, environmental factors, and individual factors can be controlled. Figure 2 displays a flowchart that depicts the process used to develop SQI. The research gaps identified via the literature review process tie in directly with the development of the inspection algorithm. The approach outlined to develop SQI is informative in proposing the methodology to implement AI-based visual inspection on the shopfloor. Figure 3 shows the different stages involved in implementing SQI in the manufacturing/production area. There are a total of six stages—from receiving the product at the inspection area to inspecting it using AI and documenting the results. The processes and steps involved in each of the stages are described below.

1. Stage 1: Manufacturing product arrives at the inspection area:
  In the first stage, the product from the assembly line is brought to the inspection area. The item is placed in a designated location to allow the inspection process to begin.
2. Stage 2: Product image is captured
  In this stage, a high-quality camera is used to capture images of the product undergoing inspection. The lighting conditions and distance from the product are measured based on the product size and camera equipment in use.
3. Stage 3: Image preprocessing
  It is identified if grayscale or color images would be appropriate based on the availability of computational resources and desired precision and accuracy of predictions. Any augmentation or transformation is done at this stage—flips, shears, rotation, shifts, whitening, contrast adjustment, etc.
4. Stage 4: CNN-based defect detection
  A custom CNN architecture is used to detect defects in images. The architecture has the versatility to handle different types of images with just a small number of changes. The model is trained on images of defective products and non-defective products to learn the necessary feature representations. The defect detection model is built into an application that can be used on the shop floor to make the inspection process trouble-free.
5. Stage 5: Decision stage—accept/reject the product
  The operator inspects the product using the defect detection algorithm and instantaneously receives the inspection results from the computer application. Based on the results, a decision is made whether to accept or reject the product.
6. Stage 6: Document results in the inspection log
  The results of the inspection process are input into the SQI shop floor application and are automatically stored in a spreadsheet.

## 8. Casting Product Dataset

**Figure 4:**
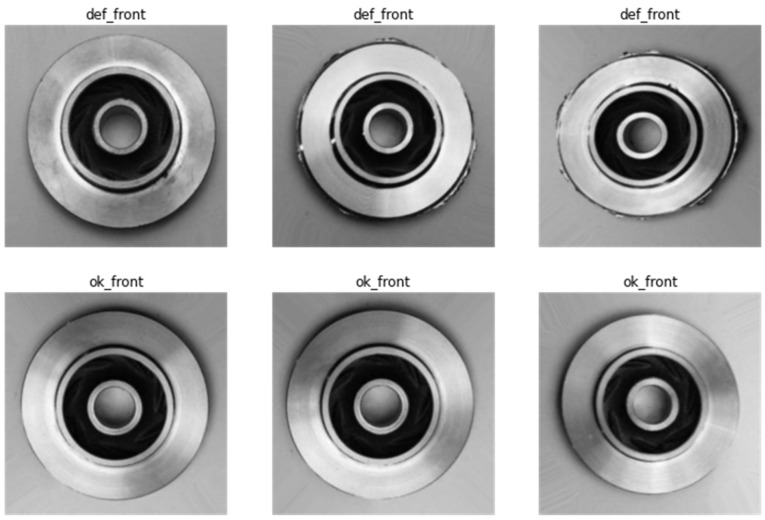
Sample images showing ‘defective’ and ‘okay’ stainless steel castings.

The dataset used in this paper is from Pilot Technocast, an SME that manufactures casting products in Gujarat, India. The data has been made available publicly by Ravirajsinh Dabhi [49]. The data consists of 7348 top-view images of submersible pump impellers. The products are made with stainless steel in a shell molding casting process. The images are captured under stable lighting using a Canon EOS 1300D camera kit produced by Canon Inc. located in Tokyo, Japan. Each image is converted to a size of 300 × 300 pixels. The data is pre-labeled into two classes: ‘def_front’ and ‘ok_front’, meaning defective and acceptable, respectively. A sample of six images that show three defective three acceptable castings are shown in Figure 4.

## 9. SQI—Modelling and Design

### 9.1. CNN Model

The AI model chosen for the inspection task is a CNN with a custom architecture. The network is constructed using a set of Convolutional Layers (Conv2D), Max Pooling Layers (MaxPooling2D), Activation Functions, and Dense Layers. The convolution operation performed by the Conv2D layer is a dot product of the ‘kernel’ and the image. The MaxPooling2D layer creates a pooled feature map by reducing the parameters involved. The max pooling layer consists of a technique called zero-padding that involves adding zeros to the edges of the image before the next convolution operation. This preserves any features that are generated at the edges of the images. In the proposed model, we use a kernel of size 3 × 3 for the convolutional layer and zero-padding in the max pooling layer. For the dense layer, we use the Rectified Linear Unit (ReLU) activation function [50]. Equation (1) shows the ReLU function where *x* is the input to the neuron.

(1) $$f\left(x\right)=\max\left(0,x\right)=\left\{\begin{array}{l} x,\;\;\mathrm{if}>0\\ 0,\;\;\mathrm{otherwise} \end{array}\right.$$

**Figure 5:**
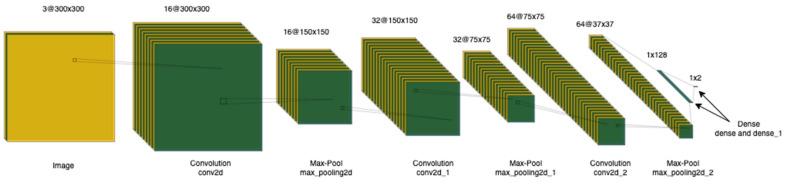
CNN architecture for Smart Quality Inspection.

The model also uses the Adaptive Moment Estimation (Adam) optimizer [51] and the sparse categorical cross-entropy loss function from Keras [52]. A summary of the model generated by the Keras library is shown in Table 3 and a visualization of the model architecture is shown in Figure 5.

A brief explanation of the different layers of the model is provided below:

1. Input Layer: The input layer is the raw image either in grayscale or Red-Green-Blue (RGB) format with (300, 300) as its dimensions. This 300 × 300 image is an array of pixels, with 300 as width and 300 as height.
2. Rescaling Operation: Neural networks generally perform better when the inputs are normalized. The channel coefficients for images are in the [0, 255] range, which is high. Higher numeric values may be computationally more expensive and could affect performance. For the casting data, we rescale the inputs to the [0, 1] range by using a 1/255 scaling factor.
3. Convolution Layer (conv2d): The first of the three convolution layers has 448 parameters with the data as (300, 300, 3) shaped array.
4. Max-Pooling (max_pooling2d): The pooling layer is useful in reducing the number of dimensions of the data. Pooling not only reduces the consumption of computing resources but also improves overall performance [53]. Max-pooling helps optimize the feature space by identifying the maximum value of elements from every pool, thereby achieving scale invariance [54].
5. Convolution Layer (conv2d_1): The second convolution layer has 4640 parameters
6. Max-Pooling (max_pooling2d_1): Like the max_pooling2d, this layer is aimed at optimizing the feature space from (150, 150, 32) to (75, 75, 32).
7. Convolution Layer (conv2d_2): The third convolution layer has 18496 parameters with an input shape of (75, 75, 64).
8. Max-Pooling (max_pooling2d_2): This max-pooling layer further reduces the dimensions of the feature map from (75, 75, 64) to (37, 37, 64) by selecting the maximum value of elements from every pool.
9. Flatten Layer (flatten): The pooled feature map is transformed from 3 dimensions to a 1-dimensional vector. This layer essentially collapses all the input into a single dimension.
10. Dense Layer (dense and dense_1): The dense and dense_1 layers from the model are geared towards the classification task. In general, a dense layer is a fully connected layer—every input and output neuron have a connection. The dense layer uses a ReLU [50] activation function and the dense_1 layer is designed with a number of output nodes equal to the number of classes.

### 9.2. Shop Floor Application

**Figure 6:**
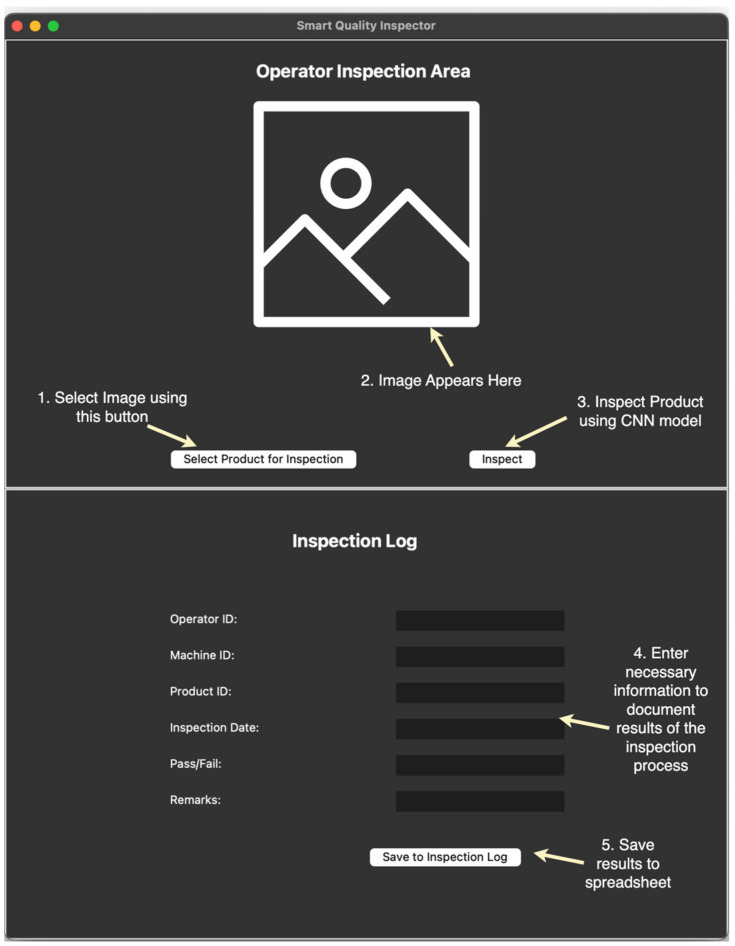
Shop floor application for Smart Quality Inspection.

The aim of this application is to enable a hassle-free inspection process on the shop floor. Images of the product from the assembly line can be uploaded into the application, and the CNN model will inspect the product to determine whether it is defective or acceptable, thereby failing or passing inspection respectively. Additionally, the SQI application allows the operator to document the findings in the inspection log. Information such as product identifiers, machine identifiers, the result of the inspection, and additional remarks can be stored in the inspection log. Figure 6 shows the SQI application window.

## 10. Performance and Results

Stages 1–3 of the SQI method involve receiving the product, capturing product images, and preprocessing images. The casting data considered in our work consists of images that were captured under stable lighting and with a Canon EOS 1300D camera [49]. Additionally, some augmentations were already applied to the image data—shear, crop, contrast adjustment, etc. The only pre-processing necessary was rescaling the images before applying the CNN model.

### 10.1. Training and Validation Performance

**Figure 7:**
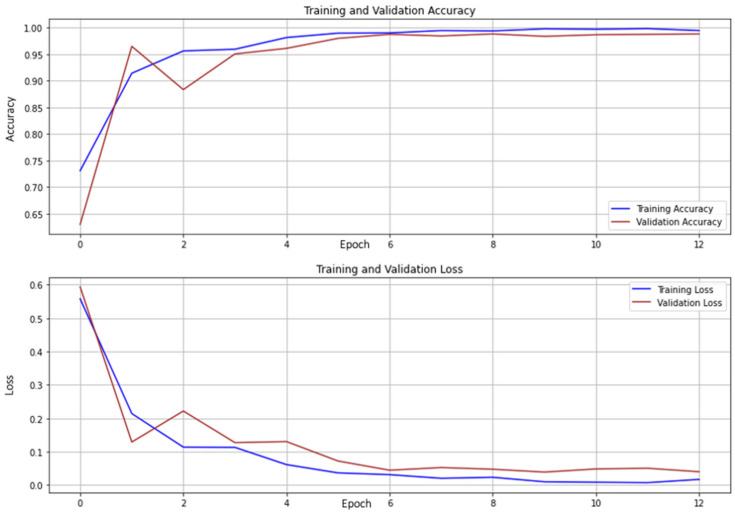
Monitoring the accuracy and loss of the training and validation set.

The training dataset consists of 6633 images, out of which 5307 images were used purely for training the model and the rest 1326 files were used in the validation set to tune the model. To optimize the performance of the model and usage of computational resources, we use the ‘Autotune’ option from the Tensorflow library. As noted previously, the ReLU activation functions were used in the Conv2D and Dense layers, and the loss function considered was a sparse-categorical cross-entropy loss. The Adam optimizer was used to compile the model, but it is worth noting that other optimization methods were also tested. The Root Mean Squared Propagation (RMSProp) and Nesterov-accelerated Adaptive Moment Estimation (Nadam) were also explored but the Adam optimizer narrowly outperformed them on the casting data. The training phase was set to run for a maximum of 20 epochs but was completed in 13 epochs. This is due to the inclusion of the early stopping criteria as a safeguard against overfitting [55]. We use validation loss as the criteria for early stopping, and the execution of the model is interrupted when the validation loss does not improve. The training and validation accuracy along with the training and validation losses have been monitored at the end of each epoch. The plot in Figure 7 shows how the accuracies and the losses of the training and validation set change by epoch.

### 10.2. Testing Results

The performance of the model is evaluated on test data of 715 images. Overall, the model achieves an accuracy of 99.86% and outperforms all the other existing models from published works. Table 4 compares the performance metrics of SQI’s proposed model with other models.

**Figure 8:**
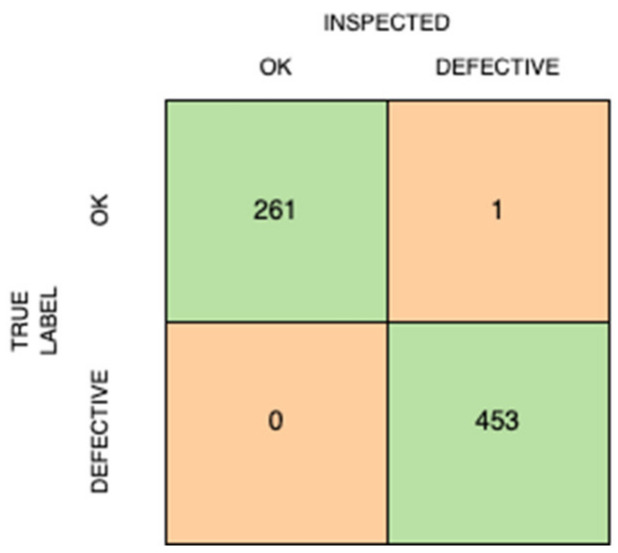
Confusion matrix showing the results of the inspection process.

We can also note that there was only one product that erroneously failed inspection when it was actually an acceptable product. If we look at the confusion matrix in Figure 8, we can see the results of the inspection on 715 images of the test set. There are 261 images that are correctly labelled as ‘OK’ (acceptable) and 453 images that are correctly labelled as ‘DEFECTIVE’ (rejected). These are the True Positive and True Negative values, respectively. On the other hand, one image that has a true label of ‘OK’ has been incorrectly classified as ‘DEFECTIVE’, resulting in one False Positive value. No images of ‘DEFECTIVE’ products were incorrectly classified—meaning no False Negative values.

In the case of quality inspection, False Positives are regarded as Producer’s risk and False Negatives are regarded as Consumer’s risk. Producer’s risk is the error of rejecting a good-quality product, and Consumer’s risk is the error of accepting a bad-quality product. In manufacturing, the aim is to reduce or minimize consumer’s risk while producer’s risk is acceptable to some extent. Based on the evaluation of 715 images, our proposed Smart Quality Inspection approach shows that there is no risk to the consumer, i.e., no defective products have been incorrectly accepted by the model.

### 10.3. Results from Shop Floor Application

**Figure 9:**
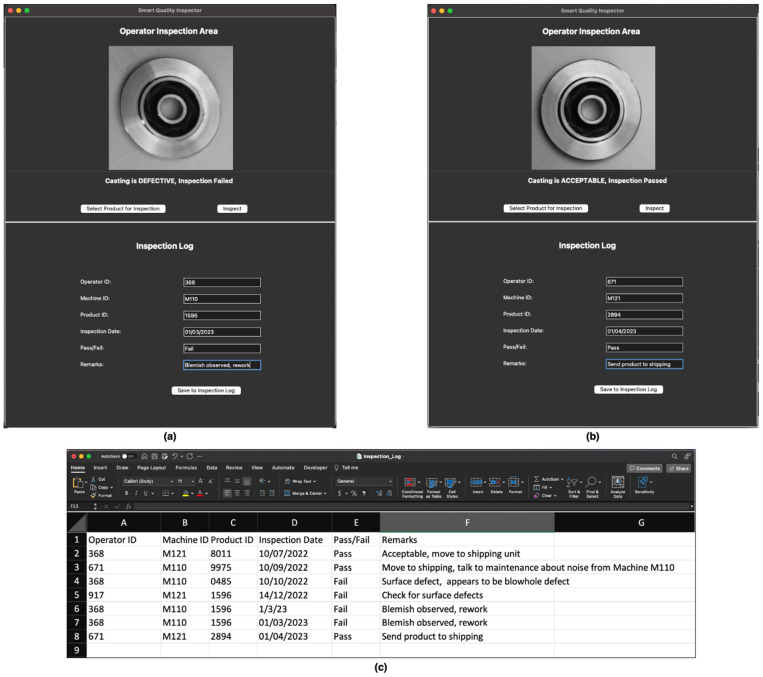
Shop floor application for Smart Quality Inspection. (**a**) shows the inspection of a defective product, (**b**) shows the inspection of an acceptable product, and (**c**) shows the results of the inspection documented in the inspection log (spreadsheet opened in Microsoft Excel).

Using the shop floor application for SQI, we can inspect the casting products. To demonstrate the application’s functionalities, we inspect a defective product and an acceptable product. Figure 9a,b shows the results of the inspection of a defective product and an acceptable product, respectively. With a literal click of a button, the product is inspected. The operator is then able to document the inspection process by entering identifying information related to the product, machinery, etc. The information entered is saved in an inspection log in the form of a spreadsheet (see Figure 9c).

## 11. Conclusions and Future Work

This paper addresses the area of product health monitoring from the perspective of the quality inspection process. The monitoring of system health and product health in manufacturing are reviewed. The steps involved in the quality inspection and visual inspection process are discussed, and key factors that affect the visual inspection process are analyzed. The casting operation is reviewed and the process of visual inspection of casting products is examined. Based on the challenges involved in visual inspection, the AI-based approach of Smart Quality Inspection (SQI) is proposed. A custom CNN model for SQI is designed and implemented on casting product images. The model achieves a high accuracy of 99.86% in inspecting casting products. The accuracy and F-1 score for the model are the highest compared to all the published works on the dataset. Additionally, a shop floor application is also developed to make the inspection process hassle-free. The goal of the application is to minimize as many factors affecting the inspection process as possible. The effects of many of the operator or individual factors, social factors, and organizational factors are minimized with AI-based inspection. Even some of the task factors and environmental factors are controlled. For instance, in an automated inspection system, environmental factors such as time of the day and shift duration would have no impact on the AI model’s performance. The application also allows the quality inspector to document their findings from the inspection process and store it in an inspection log.

**Figure 10:**
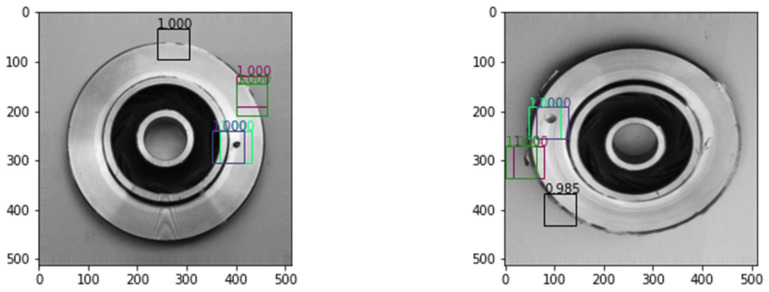
Localized defect detection with detection probability on two 512 × 512 sized images.

We do believe that there are areas in which the proposed work can be improved upon. The defects from the casting products could be classified into different types: blowholes, surface blemishes, cracks, adhesions, etc. A formal categorization of defects could be undertaken before classifying them. Localized detection of defects is another feature that can be incorporated into SQI. Localized defect detection identifies the exact location of defects and will return a probability of a defect occurring at that location. This feature is a work in progress, but at this time the results are not satisfactory. Figure 10 shows an example of localized fault detection on casting product images.

So far, the proposed SQI method has been tested on images of casting products. Some of the environmental conditions during the data collection were not in our control. For example, we had no control over the lighting setup to capture the images nor were we able to select the camera equipment used. This is the case with most publicly available datasets. On the other hand, there is limited real-time accessibility to factories where one can perform experiments. In an ideal scenario, this system can be set up directly on the assembly line by automatically taking a feed of product images. This would automate the inspection process even further and reduce any remaining factors that might affect an inspector’s performance. Data from the inspection logs of the SQI tool could also be studied using various Natural Language Processing (NLP) techniques. NLP methods are gaining importance in manufacturing, especially in maintenance.

## Figures and Tables

**Table 1:** Factors that affect visual inspection.

Task	Environmental	Operator or Individual	Organizational	Social
▪ Defect Rate ▪ Type of defect ▪ Defect detectability ▪ Location of defect ▪ Complexity of task ▪ Standards for comparison ▪ Time available to complete task ▪ Multiple inspections for each task ▪ Inspection aids ▪ Level of automation	▪ Temperature ▪ Humidity ▪ Lighting ▪ Noise ▪ Time of the day ▪ Duration of shifts ▪ Workplace ergonomics ▪ Alertness or level of vigilance	▪ Age ▪ Intellectual Aptitude ▪ Level of intelligence ▪ Gender ▪ Visual acuity ▪ Depth perception ▪ Concentration level ▪ Biases	▪ Support from management ▪ Training and retraining ▪ Incentives, bonuses ▪ Feedback on performance ▪ Job rotation	▪ Relationship with peers ▪ Communication ▪ Isolation ▪ Pressure

**Table 2:** Defect detection methods in manufacturing.

Reference	Defect Detection Method	Parameters Considered	Results
[43]	Edge-based Federated Learning	Metal Nut Data	Federated model outperforms local models in detecting defects
[44]	CNN	Welded LPG pressure vessel products	97.7% accuracy for classification task
[45]	ResNET and Fully Convolutional Network	Automated Fiber placement inspection	Raw pixel accuracies recorded using a User-Interface
[46]	CNN	Injection Molding	Accuracy of more than 90% achieved
[47]	CNN	End-milled machined surfaces	Accuracy of 92.91% in classifying roughness
[48]	YOLO and SSD models	Painting defects in shipyards	Accuracy of 90.4% and 82% using the YOLO and SSD models respectively

**Table 3:** Keras model summary.

Layer (Type)	Output Shape	Param #
rescaling (Rescaling)	(None, 300, 300, 3)	0
conv2d (Conv2D)	(None, 300, 300, 16)	448
max_pooling2d (MaxPooling2D)	(None, 150, 150, 16)	0
conv2d_1 (Conv2D)	(None, 150, 150, 32)	4640
max_pooling2d_1 (MaxPooling2D)	(None, 75, 75, 32)	0
conv2d_2 (Conv2D)	(None, 75, 75, 64)	18496
max_pooling2d_2 (MaxPooling2D)	(None, 37, 37, 64)	0
flatten (Flatten)	(None, 87616)	0
dense (Dense)	(None, 128)	11214976
dense_1 (Dense)	(None, 2)	258

Model: “sequential”. Total params: 11,238,818. Trainable params: 11,238,818. Non-trainable params: 0.

**Table 4:** Comparison of the performance of our proposed model with other models from published works.

Model	Precision	Recall	F1 Score	Accuracy
CNN with Densenet [56]	99.08%	100%	99.54%	99.42%
EfficientNetB0 [57]	97.11%	95.87%	-	96.88%
CNN-based Vision System [58]	-	-	-	99.7%
Transfer Learning with DenseNet [59]	97.96%	95.58%	-	95.94%
CNN model for Holonic Shop Floor [41]	-	-	-	99.82%
VGG-16 with CNN [60]	98.7%	94.1%	-	95.8%
Vision Transformer [61]	99.66%	99.33%	-	99.58%
Accelerated CNN [62]	99.24%	100 %	99.62%	99.72%
Proposed Smart Quality Inspection (SQI) Model	99.62%	100%	99.81%	99.86%

## Data Availability

The data used in this research is publicly available at https://www.kaggle.com/datasets/ravirajsinh45/real-life-industrial-dataset-of-casting-product [acessed 10 January 2023].
